# Biological Control and Growth-Promoting Potential of the Endophytic Fungus *Nigrospora sphaerica* Against Anthracnose in *Begonia benariensis*

**DOI:** 10.3390/jof12060412

**Published:** 2026-06-05

**Authors:** Shuwen Liu, Mian Liu, Jian Liu, Huali Li, Yajiao Sun, Mengyao Wang, Hongliang Zhang, Yunqiang Ma, Junjia Lu

**Affiliations:** 1 College of Landscape Architecture and Horticulture Sciences, Southwest Forestry University Sciences, Kunming 650224, China; 15887642939@163.com (S.L.); 18311782998@163.com (M.L.); jian927520@163.com (J.L.); 15912938064@163.com (H.L.); 18087323192@126.com (Y.S.); 17587020327@163.com (M.W.); 2 Yunnan Key Laboratory of Landscape Plant Resource Cultivation and Application, Southwest Forestry University, Kunming 650224, China; mayunqiang@swfu.edu.cn; 3 Guizhou Academy of Sciences, Guiyang 550001, China; zhl69827@sina.com

**Keywords:** *Begonia benariensis*, anthracnose, *Nigrospora sphaerica*, biological disease control, growth-promoting function

## Abstract

To explore efficient and sustainable biocontrol resources against anthracnose in *Begonia benariensis*, endophytic fungi were isolated from healthy host tissues and screened for antagonistic activity against *Colletotrichum aotearoa* SWBG5. Among 31 isolates, four showed strong inhibition, and the most potent strain, QYN6, exhibited an in vitro mycelial inhibition rate of 63.67%. Based on morphology and multi-gene phylogeny (*ITS*, *TUB2*, *TEF-1α*), QYN6 was identified as *Nigrospora sphaerica*. Mechanistic assays revealed that QYN6 secretes multiple cell wall-degrading enzymes (chitinase, β-1,3-glucanase, cellulase, protease) and displays hyperparasitism against the pathogen hyphae (entwining, deformation, swelling), acting synergistically to inhibit fungal growth. In greenhouse pot trials, QYN6 achieved a biocontrol efficacy of 48.91% against *Begonia* anthracnose. Additionally, QYN6 significantly activated host defense responses, increasing the activities of antioxidant enzymes (SOD, POD, PPO, CAT) and the contents of soluble protein and soluble sugar. Furthermore, QYN6 exhibited multiple plant growth-promoting traits, including IAA production, siderophore synthesis, and potassium solubilization. Inoculation with QYN6 markedly improved plant height, leaf number, root length, and biomass of *B. benariensis*. Overall, *N. sphaerica* QYN6 possesses dual biocontrol and growth-promoting potential, providing a promising microbial resource and theoretical basis for green management of *Begonia* anthracnose.

## 1. Introduction

*Begonia benariensis* is a perennial herbaceous flower belonging to the genus *Begonia* L. [1]. It boasts advantages such as vivid flower colors, a long flowering period, and the ability to grow normally in full sunlight or semi-shaded environments [2]. Owing to its excellent ornamental properties, it possesses significant horticultural value.

Anthracnose, caused by fungi of the genus *Colletotrichum*, is a globally distributed plant fungal disease [3]. In 2012, *Colletotrichum* species were recognized as the eighth-most important group of plant pathogenic fungi globally [4]. This genus has an extremely broad host range, as it can not only infect field crops and cash crops, leading to yield reduction [5], but also harm ornamental horticultural plants and medicinal plants, resulting in severe economic losses [6,7]. Anthracnose in *Begonia* primarily affects the stems and leaves. In the early stage of infection, stem lesions appear as light brown fusiform spots, which later turn brown and become sunken. In the advanced stage, numerous small black dots form densely on the lesion surface. For infected leaves, damage mostly initiates from the leaf margins, initially presenting as irregular brown spots. As the spots expand, the leaves gradually wither and eventually die completely, severely impairing their ornamental value [8]. *B*. *benariensis* is also affected by this disease [9]. However, systematic research on diseases of *B. benariensis* remains relatively scarce.

Biological control using endophytes as biocontrol agents has become a major research focus. For instance, endophytic fungi isolated from healthy grape leaves belong to eight genera including *Nigrospora*, *Alternaria* and *Fusarium*. Dual-culture assays confirmed that six strains significantly inhibited the mycelial growth of *Colletotrichum gloeosporioides*, the causal agent of grape anthracnose. Further investigation demonstrated that strains S5 and MM4 could secrete volatile organic compounds with antifungal activity [10]. Sodhi et al. [11] found that endophytic *Nigrospora oryzae* could stably colonize rice tissues. The strain exhibits excellent plant growth-promoting traits, including IAA production and phosphorus solubilization. Under saline–alkali stress, plants treated with this endophytic fungus showed increases of 48.39%, 30.94%, 25.32% and 43.67% in leaf relative water content, chlorophyll, phenolic and osmolyte contents compared with the control group. Similar variation trends were also observed under drought stress. The results demonstrated that *N. oryzae* could effectively alleviate abiotic stress damage and maintain normal growth of rice plants under adverse environments. Nifakos et al. [12] isolated a variety of endophytic fungi as well as the pathogenic fungus *Colletotrichum scovillei* from symptomless olive trees. Further bioassays confirmed that several of the isolated endophytic strains exhibited strong antagonistic activity against *Colletotrichum* pathogens, highlighting their great potential as novel biocontrol resources for anthracnose management. Liu et al. [13] obtained endophytic fungus COTG-19 from healthy camellia roots, which showed strong antagonism against multiple *Colletotrichum* species with an inhibition rate up to 84%. Leaf inoculation assays confirmed its excellent preventive and curative effects on camellia anthracnose. The strain degrades pathogen cell walls via lytic enzymes, and releases volatile organic compounds to suppress mycelial growth, spore germination and appressorium formation as well as to damage cell membrane integrity. It also presents stable colonization capacity and satisfactory fungicide tolerance. Nandini et al. [14] explored the induced systemic resistance (ISR) triggered by *Trichoderma harzianum* and *T. asperellum* against pepper anthracnose pathogen *C. truncatum*. Pot experiments verified that pretreatment with the two strains activated host ISR. The resistance was manifested as markedly elevated activities of five defense-related enzymes including peroxidase (POD), polyphenol oxidase (PPO), phenylalanine ammonia-lyase (PAL), catalase (CAT) and superoxide dismutase (SOD), alongside accumulation of phenolic compounds and β-1,3-glucanase.

Biocontrol endophytic fungi prevent pathogen invasion and restrict growth via niche occupation and competing for nutrients and trace elements [15,16]. They secrete cell wall-degrading enzymes (CWDEs) such as β-1,3-glucanase and chitinase to degrade pathogen cell wall components, and also parasitize pathogens by entangling, penetrating, lysing, and inhibiting hyphae, thereby invading and inactivating or killing them [17]. These combined actions effectively inhibit pathogen growth and reproduction [18,19]. In addition, endophytic fungi can activate the plant defense system, namely induced systemic resistance (ISR), and promote plants to produce more defensive enzymes to resist diseases [20,21], and at the same time stimulate plant development processes and improve the environmental adaptability of plant hosts by virtue of their abilities such as phosphorus solubilization, potassium solubilization, siderophore production, and secretion of indole-3-acetic acid (IAA) [22,23].

This study screens out antagonistic strain QYN6 from healthy *B. benariensis* tissues and conducts systematic identification of the isolate. Multiple evaluations are carried out to clarify its biocontrol and growth-promoting potentials, covering antagonistic activity against *C. aotearoa*, secretion of functional metabolites, host defense response regulation and plant growth improvement performance.

## 2. Materials and Methods

### 2.1. Materials

The test included healthy *B*. *benariensis* plants (for endophytic fungus isolation) and 1-month-old *B. benariensis* seedlings (8–11 cm tall) purchased from Foshan Nanhai Jincaidie Horticulture Co., Ltd., Foshan, Guangdong, China (for assessing induced resistance and growth-promoting effects).

The test pathogen was *C*. *aotearoa* SWBG5, a strain with the strongest pathogenicity isolated and screened, which is preserved in the Laboratory of College of Landscape Architecture and Horticulture, Southwest Forestry University, Kunming, Yunnan, China.

Culture media and reagents used in the experiment included potato dextrose agar (PDA), qualitative culture media for chitinase, β-1,3-glucanase, protease and cellulase, quantitative culture media for chitinase, β-1,3-glucanase, protease and cellulase, King’s B medium, organic phosphorus yolk medium, inorganic phosphorus medium, chrome azurol S (CAS) medium, iron-free Czapek medium and potato dextrose broth (PDB). The detailed formulas of all media and reagents are listed in Supplementary Table S1.

### 2.2. Isolation of Endophytic Fungi and Determination of Antifungal Rate

Collected healthy *B. benariensis* plants were rinsed with running water to remove surface contaminants and continuously washed for 1 h to eliminate epiphytic microorganisms, improving surface disinfection efficiency. Disinfection duration was adjusted according to plant tissue features and disinfectant concentration. Subsequent surface disinfection was conducted in a laminar flow hood: roots, stems, and leaves were soaked in 75% ethanol for 2 min, 1 min, and 30 s respectively, then rinsed 3–5 times with sterile water. Subsequently, they were immersed in 5% sodium hypochlorite solution for 3 min (roots), 2 min (stems), and 45 s (leaves), and finally rinsed with sterile water 5–8 times. Sterilization efficiency was verified by spreading 100 μL of the final rinsing water onto PDA plates. No colony growth confirmed complete removal of epiphytic microbes.

After disinfection, endophytic fungi were isolated via the tissue separation method [24]. Tissue blocks of approximately 0.5 cm × 0.5 cm were cut, ten replicate plates were prepared for roots, stems and leaves separately, with a total of 150 tissue specimens used in the experiment. The cultures were incubated upside down in a constant-temperature incubator at 25 °C for approximately 7 days. Edge mycelia were picked for purification to obtain contamination-free colonies.

Determination of the inhibition rate against *C. aotearoa* [25]: 5 mm-diameter pathogen plugs were excised with a cork borer (Solarbio, Beijing, China) and inoculated onto PDA plates. Endophytic fungi were inoculated 2 cm from the center in a cross pattern, with 3 replicates each. Pathogens cultured alone served as the control. After one week of cultivation, the diameter of the pathogen in the control group (D) and the treatment group (d) were measured using the cross method. The inhibition rate was calculated according to the formula: “Inhibition rate (%) = [(D − d)/(D − Db)] × 100%” (where Db is the initial diameter of the pathogen plug, 5 mm).

### 2.3. Identification of Endophytic Fungi

#### 2.3.1. Morphological Identification

The endophytic fungus with the strongest antimicrobial activity, a vigorously growing strain, was inoculated onto PDA medium and cultured in a constant-temperature incubator at 25 °C. Colony diameter was measured and morphological characteristics were recorded at 24 h intervals. Further observations were conducted using the slide culture method: a piece of Czapek medium (1 mm thick, 1 cm × 1 cm) was placed on a glass slide, and mycelia of the culture were picked with a sterilized inoculating needle and inoculated onto one side of the medium. A coverslip was then placed over the culture to prepare a moist-chamber slide culture. Following 3–5 days of incubation, the strain’s conidia, conidiophores, and conidiogenous cells were observed and photographed using an Olympus CX33 microscope (Olympus Corporation, Tokyo, Japan). Adobe Photoshop 2025 (version 25.0, Adobe Inc., San Jose, CA, USA) was applied for subsequent digital image processing and analysis [26,27].

#### 2.3.2. Molecular Biological Identification

Based on the genus name of the screened endophytic fungus, primers *ITS*, *TUB2*, and *TEF-1α* were selected (Table 1). Reaction systems were prepared (Table 2 and Table 3), and PCR amplification programs established (Table 1) to amplify preserved endophytic fungal DNA sequences. The isolated endophytic fungal strain has been deposited in the strain library of the College of Landscape Architecture and Horticulture, Southwest Forestry University, Kunming, Yunnan, China. Following gel electrophoresis detection, amplification products were sent to Shanghai Sangon Biotech Co., Ltd., Shanghai, China for sequencing. Sequencing results were submitted to NCBI’s GenBank database (https://www.ncbi.nlm.nih.gov/genbank/; accessed on 20 April 2026) to obtain accession numbers, with subsequent BLAST alignment (https://blast.ncbi.nlm.nih.gov/Blast.cgi; accessed on 20 April 2026) of the sequencing results. Sequences of strains with high homology were downloaded, and different gene fragments of the same strain were spliced. A phylogenetic tree was built using the Neighbor-joining method in MEGA 11.0 (Molecular Evolutionary Genetics Analysis Version 11), with 1000 bootstrap replicates.

### 2.4. Observation of Hyperparasitism Phenomenon of Endophytic Fungi

In a laminar flow hood, mycelia of the pathogen were picked with a sterile inoculating needle and placed on one side of the PDA medium, and the endophytic fungus was placed on the other side, with 3 replicates. Once the mycelia of the endophytic fungus and pathogen made contact, contact-point hyphae were collected daily and transferred to glass slides. A coverslip was placed on the slide, followed by dropwise addition of sterile water, and the mycelial interactions between the endophytic fungus and the pathogen were observed under a microscope.

### 2.5. Detection of CWDEs Produced by Endophytic Fungi

#### 2.5.1. Qualitative Detection

5 mm diameter mycelial disks of endophytic fungi were punched using a sterile cork borer and inoculated onto selective qualitative media. Synchronously, blank controls were established by placing sterile PDA disks with uniform size and shape in the absence of fungal inoculum. All plates were incubated inversely at a constant temperature of 28 °C. After 3 days of cultivation, the emergence of transparent hydrolysis zones was examined for qualitative determination of chitinase, β-1,3-glucanase, cellulase, and protease activities [28,29,30].

#### 2.5.2. Quantitative Determination

Series of standard solutions of varying concentrations for N-acetylglucosamine, glucose, and tyrosine were prepared. Absorbance was measured via a Tu-1901 double-beam UV-visible spectrophotometer (Beijing Purkinje General Instrument Co., Ltd., Beijing, China) spectrophotometer: 540 nm for chitinase, β-1,3-glucanase, and cellulase, and 680 nm for protease. Standard curves were plotted, and their equations derived. The measured OD values were substituted into the standard curve equations, and calculations were performed using the following formula. Detailed methods are provided in the Supplementary Materials.

Enzyme activity (U·mL^−1^·min^−1^) = (X × V_total × 1000)/(V_sample × T)

Note: X represents the content of reducing sugar calculated by substituting the absorbance value of the sample into the standard curve formula; V_total represents the total volume of the sample measured; 1000 represents the conversion factor (1 mg = 1000 μg); V_sample represents the volume of enzyme solution used for measurement; T represents the reaction time.

### 2.6. Biocontrol Efficacy of Biocontrol Fungi

In vitro biocontrol efficacy: Following the methods described by Holkar et al. [10] and Wu et al. [31], fungal spore suspensions at a concentration of 1 × 10^7^ CFU·mL^−1^ were prepared with sterile distilled water. Leaves of *B. benariensis* were surface-sterilized and wounded, then inoculated with pathogen spore suspension (30 μL per wound, one drop per site). After 24 h, a spore suspension of the biocontrol strain QYN6 was applied to the wounded sites. Leaves treated with sterile water alone served as the control. Each treatment was performed with three biological replicates. The leaves were incubated at 28 °C under constant humid conditions for 7 days. Subsequently, lesion areas were measured to calculate biocontrol efficacy and disease index. The disease grading criteria referred to the method reported by Sun et al. [32], with minor modifications according to Willocquet et al. [33]. The specific classification standards were defined as follows: grade 0 with no lesion area, grade 1 for lesion area ranging from 0.1% to 1%, grade 2 for 1% to 5%, grade 3 for 5% to 10%, and grade 4 for lesion area exceeding 10%. The calculation formula is as follows:

Disease index = [Σ (number of diseased leaves at each grade × value of the corresponding grade)]/[total number of leaves surveyed × highest grade value].

Biocontrol efficacy (%) = (disease index of the control group − disease index of the treatment group)/disease index of the control group × 100%.

Potted plant biocontrol efficacy: The experiment was carried out in a greenhouse, with controlled environmental conditions: day/night temperature of 30 °C/23 °C, relative humidity of 70–80%, photoperiod of 12 h light/12 h dark, and regular light intensity of 300–400 μmol·m^−2^·s^−1^. All potted plants were watered once every two days with a fixed volume of sterile water to maintain consistent soil moisture [34]. *B. benariensis* plants were individually transplanted into pots (13 cm × 15 cm) containing 1300 g sterilized soil, with six pots per treatment. Spore suspensions of pathogen and endophytic fungi were prepared with sterile water at a concentration of 1 × 10^7^ CFU·mL^−1^.

Foliar spray inoculation was adopted: 20 mL of SWBG5 spore suspension was sprayed first, followed by 20 mL of QYN6 spore suspension after 24 h. Sterile water was used as the control, with 3 replicates for each treatment.

After 7 days of incubation, the biocontrol efficacy and disease index were calculated using the same method as described above.

### 2.7. Induced Resistance of Endophytic Fungi to Plants

#### 2.7.1. Greenhouse Potting Treatment

In a laminar flow hood, sterile cork borers extracted endophytic fungal and pathogenic plugs, which were subsequently inoculated into sterilized medium at 3 plugs per 100 mL of medium. Cultures were incubated on a constant-temperature shaker (MQW‑63R, Shanghai Minquan Instruments Co., Ltd., Shanghai, China) at 28 °C and 200 rpm for 168 h, followed by fungal suspensions preparation via mechanical crushing and spore concentration was adjusted to 1 × 10^7^ CFU·mL^−1^ using a hemocytometer (XB-K-25, Shanghai Qiujing Biochemical Reagent and Instrument Co., Ltd., Shanghai, China).

*B. benariensis* plants with similar growth vigor were selected and treated by irrigating 100 mL of liquid via the root-injury irrigation method. Specifically, uniform and slight root damage was artificially created on the root surface of each plant using a sterile needle to simulate natural root wound. This treatment facilitates stable colonization of endophytic fungi and pathogen infection in root tissues [35]. Four treatments were established: water irrigation control (CK), pathogen suspension alone (CA), endophytic fungal suspension alone (QYN6), and endophytic fungal suspension 24 h post-pathogen suspension irrigation (QYN6 + CA), with 3 replicates per treatment. The activities of plant defense enzymes, as well as the contents of soluble protein and soluble sugar, were measured at 1 d, 3 d, 5 d, 10 d, 15 d, and 30 d after treatment. For sampling, the first leaf near the root was selected.

#### 2.7.2. Determination of Activities of Several Defense Enzymes and Contents of Soluble Sugar and Soluble Protein

Fresh leaf samples were used for all measurements. The activities of superoxide dismutase (SOD), peroxidase (POD), polyphenol oxidase (PPO), and catalase (CAT) were determined spectrophotometrically following standard protocols [36]: SOD activity was assayed by the nitroblue tetrazolium (NBT) photoreduction method, measuring the inhibition of NBT reduction at 560 nm. POD activity was determined using guaiacol as substrate, recording the increase in absorbance at 470 nm. PPO activity was assayed with catechol as substrate, monitoring the increase in absorbance at 410 nm. CAT activity was measured by monitoring the decomposition of H_2_O_2_ at 240 nm. Soluble protein content was determined by the Coomassie Brilliant Blue G-250 method, and soluble sugar content was measured by the anthrone-sulfuric acid method. Detailed procedures, including buffer compositions, reaction mixtures, centrifugation conditions, and calculation formulas, are provided in the Supplementary Materials. All assays were performed with three biological replicates.

### 2.8. Determination of Growth-Promoting Functions of Endophytic Fungi and Their Growth-Promoting Effects on Plants

#### 2.8.1. Determination of Phosphorus-Solubilizing Ability

A 5 mm-diameter plug of endophytic fungi was obtained using a sterile cork borer and inoculated onto the organic phosphorus egg yolk medium. Cultures were incubated upside down in a constant-temperature incubator at 28 °C, with 3 replicates. After 5 days, the formation of transparent zones was observed; if a transparent zone was formed, the strain was considered to have phosphorus-solubilizing ability [37].

#### 2.8.2. Determination of Potassium-Solubilizing Ability

A 5 mm-diameter plug of endophytic fungi was obtained using a sterile cork borer and inoculated onto one end of PDA medium, while the other end was evenly sprinkled with sterilized potash feldspar powder. Cultures were incubated upside down in a constant-temperature incubator at 28 °C, with 3 replicates. After 7 days, the growth characteristics and chemotaxis of the strain on the medium were observed. Strain growing towards and covering the potash feldspar powder were considered to possess potassium-solubilizing ability; those growing away from the powder lacked this ability [38].

#### 2.8.3. Determination of Siderophore-Producing Ability

Qualitative determination: A 5 mm-diameter plug of endophytic fungi was obtained using a sterile cork borer and inoculated onto CAS medium. Cultures were incubated upside down in a constant-temperature incubator at 28 °C, with 3 replicates. After 5 days, formation of brown or yellow halos was observed (Once dissolved in the medium, CAS solution forms a blue complex with CTAB; degradation of this complex results in a yellow or brown halo around the colony). Halo formation indicated the strain possessed siderophore-producing ability [39].

#### 2.8.4. Detection of IAA-Producing Ability

Qualitative determination: A 5 mm plug of endophytic fungi was obtained using a cork borer and inoculated into King’s B medium. Cultures were incubated with constant-temperature shaking at 28 °C and 200 rpm for 120 h. Strain fermentation broth was centrifuged at 8000 rpm for 15 min. After taking 2 mL of the supernatant, the strain fermentation broth was mixed with Salkowski’s color reagent at a volume ratio of 1:1, followed by a dark reaction at room temperature for 30 min. Color change was observed, with a pink color indicating IAA production and deeper color representing a higher yield.

Preparation of IAA standard curve: A series of working solutions (0.5, 1.0, 5.0, 10.0, 15.0, 20.0, and 25.0 μg/mL) were prepared by diluting a 100 μg/mL IAA stock solution. For each concentration, 2 mL of the working solution was added to a clean test tube, followed by 4 mL of Salkowski’s reagent. After 30 min of dark incubation at room temperature, absorbance measured at 530 nm. A standard curve was plotted with absorbance as the ordinate and IAA concentration as the abscissa. Determination of IAA content: Quantitative analysis was performed on color-developed samples from the qualitative assay, with 3 replicates. The absorbance at 530 nm was measured using a spectrophotometer. The obtained absorbance values were substituted into the IAA standard curve to calculate the IAA concentration of each strain. A higher value indicated a higher IAA content per mL of fermentation broth and a stronger IAA-producing ability [40].

#### 2.8.5. Determination of Growth Indicators

Endophytic fungal suspension was prepared as described in 2.7.1. Each pot was irrigated with 100 mL of an endophytic fungal spore suspension (1 × 10^7^ CFU·mL^−1^), while the control group received an equivalent volume of sterile water. Each treatment included 3 replicates. Plant height and leaf number were measured on the treatment day; after 30 days, the following parameters were determined: shoot fresh and dry weight, root fresh and dry weight, plant height, leaf number, and root length [41].

### 2.9. Data Analysis

Data were analyzed using Microsoft Excel 2021 and SPSS 25.0. Statistical significance among samples was determined using one-way analysis of variance (ANOVA) with *p* < 0.05, followed by Duncan’s multiple range test. Data visualization was performed using Adobe Photoshop 2025.

## 3. Results

### 3.1. Antifungal Effect of Endophytic Fungi

**Figure 1:**
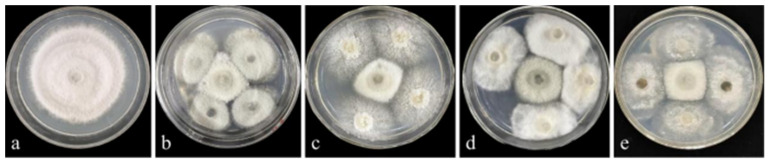
Inhibitory effects of endophytic fungal strains against pathogen SWBG5. (**a**) Control (pathogen only). (**b**) Inhibitory effect of strain QJN4. (**c**) Inhibitory effect of strain QYN6. (**d**) Inhibitory effect of strain QYN9. (**e**) Inhibitory effect of strain QYN12.

A total of 31 endophytic fungal strains were isolated from healthy *B*. *benariensis* plants. Among them, four strains exhibited strong antagonistic activity against pathogen SWBG5 and showed no pathogenicity to host plants, namely QJN4, QYN6, QYN9, and QYN12 (Figure 1). Strain QYN6 displayed the highest inhibition rate, reaching 63.67% (Table 4).

### 3.2. Identification of QYN6

#### 3.2.1. Morphological Identification of QYN6

**Figure 2:**
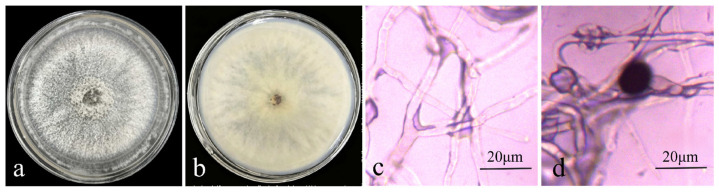
Morphological characteristics of QYN6. (**a**) Colony grown on PDA medium for 5 days. (**b**) Reverse morphology of colonies cultured on PDA medium for 5 days. (**c**) Mycelia. (**d**) Conidiophores, conidiogenous cells, conidia.

Endophytic fungus QYN6 began to grow white mycelia on the first day of cultivation, with uniform diffuse growth. It grew rapidly on the 2nd and 3rd days, and covered the Petri dish within 5–7 days of cultivation at a constant temperature of 25 °C, showing a relatively fast growth rate on PDA medium. The colony was odorless, overall slightly rough, and wooly. Initially, both the colony surface and reverse were white; as cultivation time extended, the mycelial color deepened to grayish-black, and the reverse turned dark brown to grayish-black (Figure 2a,b). The mycelia were transparent to light brown and septate (Figure 2c). Conidiophores arose from the mycelia; conidiogenous cells were flask-shaped, ampoule-shaped to subcylindrical. Conidia were non-septate, dark brown to black, smooth, and droplet-shaped or round, with dimensions ranging from (11.03–11.72) μm × (9.66–10.34) μm (Figure 2d). Based on the above morphological characteristics, QYN6 was preliminarily identified as a member of the genus *Nigrospora* [42].

#### 3.2.2. Molecular Biological Identification of QYN6

**Figure 3:**
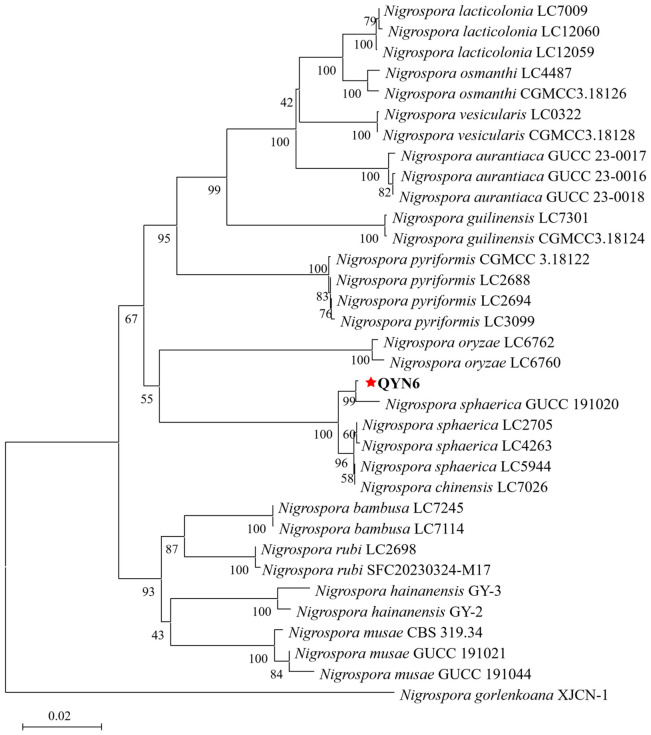
Multi-gene phylogenetic tree of QYN6. The red star indicates strain QYN6 (*Nigrospora sphaerica*).

For strain QYN6, gene fragments with lengths of 557 bp, 424 bp, and 281 bp were obtained after amplification with different primers. These fragments were submitted to GenBank, yielding accession numbers PV336075, PV363142, and PV363145. Following BLAST alignment, corresponding reference sequences were selected and downloaded. The reference sequences were concatenated in the order of ITS-*TUB2*-*TEF-1α*, and a multi-gene phylogenetic tree was constructed using MEGA 11.0 (Figure 3). Strain QYN6 and *N*. *sphaerica* GUCC 191020 clustered on the same branch in this tree, with a bootstrap support value of 99%. Integrating the results of morphological and molecular identification, the endophytic fungus QYN6 was identified as *N*. *sphaerica*.

### 3.3. Observation of Hyperparasitism by QYN6

**Figure 4:**
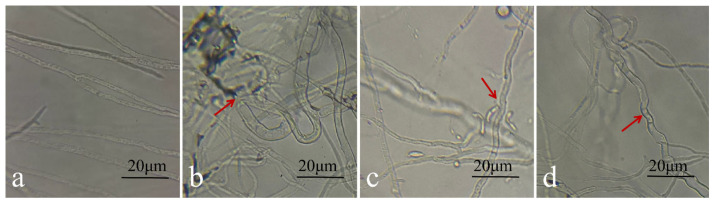
Effects of QYN6 on SWBG5. (**a**) Normal pathogen mycelia. (**b**–**d**) Pathogen mycelia under the action of QYN6. Scale bar = 20 μm.

After the endophytic fungus QYN6 was applied to the pathogen, significant changes in the mycelia were observed at 72 h post-contact. Compared with normal pathogen mycelia (Figure 4a), confrontation culture between QYN6 and the pathogen induced deformation and folding of the pathogen mycelia, along with dissolution, fragmentation, bending, and partial swelling (Figure 4b–d). These observations indicate that the endophytic fungus QYN6 exerts a hyperparasitic effect on the pathogen.

### 3.4. Results of CWDEs Produced by QYN6

**Figure 5:**
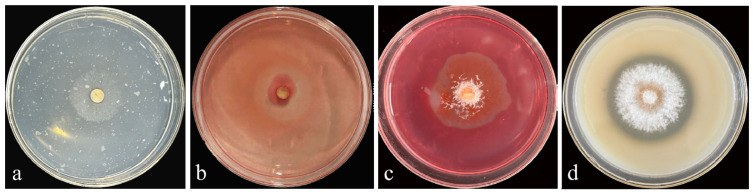
Panels (**a**–**d**) show the plate assays of chitinase, β-1,3-glucanase, cellulase, and protease produced by QYN6 in sequence.

As shown in Figure 5, no obvious transparent zone was observed for QYN6 on the chitin plate; however, the quantitative assay yielded a value of 70.85 U·mL^−1^, indicating that it has detectable chitinase production. In the qualitative assay for β-1,3-glucanase activity, an obvious transparent zone was observed on the plate, indicating a strong capacity for glucanase secretion, with a glucanase activity of 691.54 U·mL^−1^. In the cellulase assay, QYN6 exhibited a cellulase activity of 160.77 U·mL^−1^, with an obvious transparent zone formed on the plate. In the protease assay, an obvious transparent zone was observed, with the quantitative assay showing a protease activity of 233.29 U·mL^−1^.

### 3.5. Biocontrol Efficacy of Strain QYN6

**Figure 6:**
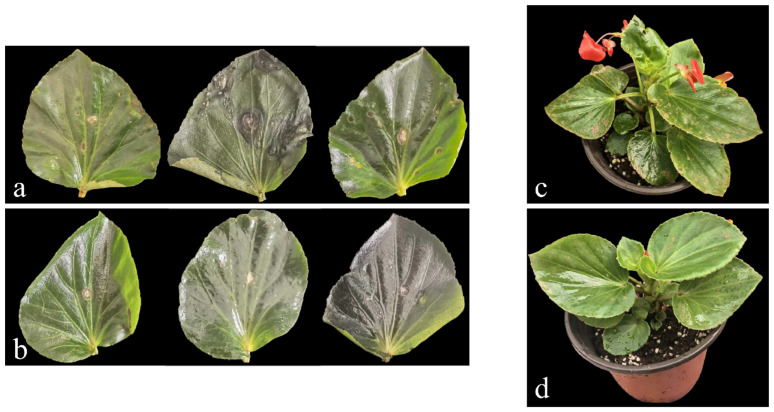
Biocontrol efficacy of strain QYN6 on in vitro leaves and in vivo potted plants. (**a**,**b**) In vitro leaf assay: (**a**) control, (**b**) QYN6 treatment. (**c**,**d**) Potted plant assay: (**c**) control, (**d**) QYN6 treatment.

As shown in Figure 6 and Table 5, for sterilized and wounded healthy leaves of *B. benariensis,* the disease index of leaves inoculated with the spore suspension of strain QYN6 at 24 h after SWBG5 inoculation had a disease index of 41.67, which was lower than that of the control group (72.22), with a control effect of 42.69%. In the potted plant assay, the disease index of plants treated with QYN6 spore suspension was 16.25, significantly lower than the control value of 31.81, and the corresponding disease control efficacy was 48.91%. Although slight data fluctuations existed due to individual differences among plants, the results confirmed that strain QYN6 possessed a certain biocontrol potential against *B. benariensis* anthracnose.

### 3.6. Induced Resistance of QYN6 to Plants

**Figure 7:**
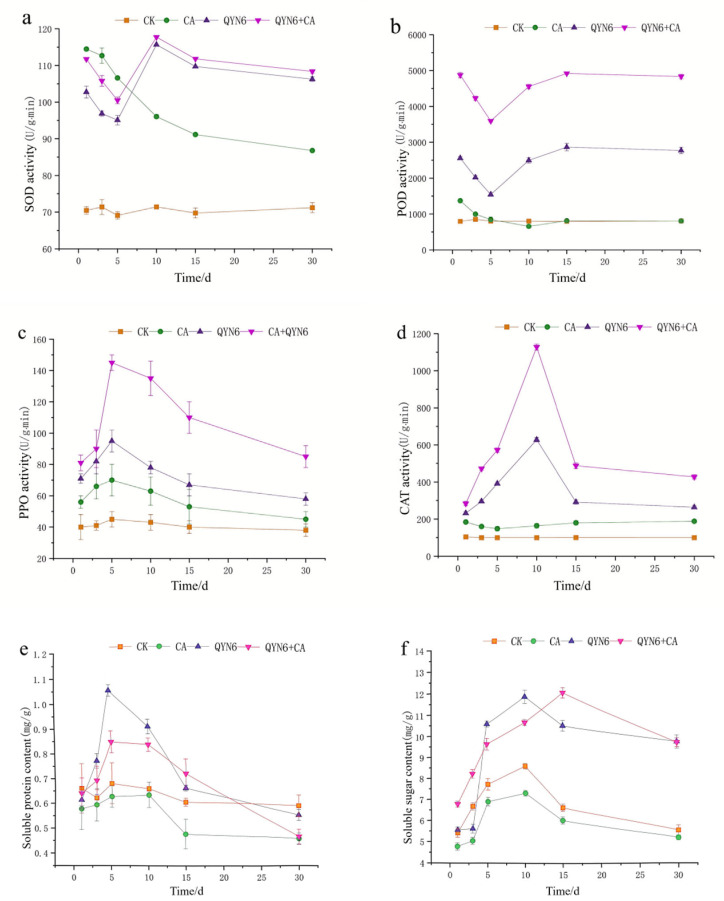
Effects of endophytic fungal strain QYN6 on the activities of four defense-related enzymes, soluble protein content, and soluble sugar content in *Begonia benariensis* leaves. (**a**) SOD activity; (**b**) POD activity; (**c**) PPO activity; (**d**) CAT activity; (**e**) Soluble protein content; (**f**) Soluble sugar content. CK: untreated control; CA: pathogen-only control (inoculated with *Colletotrichum aotearoa* alone); QYN6: endophyte-only treatment (inoculated with strain QYN6 alone); QYN6 + CA: combined treatment (inoculated with strain QYN6 and challenged with *Colletotrichum aotearoa*); SOD: superoxide dismutase; POD: peroxidase; PPO: polyphenol oxidase; CAT: catalase.

It was clearly observed that the defense enzyme activities in the CA treatment group were only slightly higher or lower than those in the CK group. In contrast, the QYN6 and QYN6 + CA treatment groups exhibited significantly higher defense enzyme activities than the CK group at most time points. The variation trend of defense enzyme activities in the QYN6 + CA group presented an initial decrease followed by an increase, or an initial increase followed by a decrease, which was similar to that of the QYN6 group. The CK group maintained the lowest and relatively stable defense enzyme activity levels (Figure 7a–d), with minor fluctuations that are likely attributable to wounding-induced stress during the experimental setup, rather than treatment-specific effects. These results indicated that strain QYN6 could enhance plant defense enzyme activities and alleviate the decline in defense enzyme activity induced by pathogenic fungi, thereby improving the disease resistance of host plants.

The soluble protein content of *B. benariensis* under different treatments generally showed a trend of first increasing then decreasing. Within 30 days, the soluble protein content in the CA treatment group was consistently lower than that in the control group. The peak soluble protein content in the CK group was 0.68 mg·g^−1^. The soluble protein content in the QYN6 treatment group was lower than that in the QYN6 + CA treatment group on the 1st and 15th days, and both reached the peak on the 5th day. The peak value of soluble protein in the QYN6 treatment group was 1.05 mg·g^−1^, which was 1.54 times that of the CK group, while the peak value in the QYN6 + CA treatment group was 0.85 mg·g^−1^, 1.25 times that of the CK group (Figure 7e). The soluble sugar content of *B. benariensis* in different treatment groups all showed a trend of first increasing then decreasing. Within 30 days after pathogen inoculation, the soluble sugar content in all treatment groups was lower than that in the control group. The peak soluble sugar content in the control group was 8.64 mg·g^−1^. The soluble sugar content in the QYN6 treatment group was consistently higher than that in the CA treatment group, with a peak value of 11.83 mg·g^−1^, which was 1.37 times that of the control group, while the peak value in the QYN6 + CA treatment group was 12.03 mg·g^−1^, 1.39 times that of the control group (Figure 7f). This result demonstrated that strain QYN6 markedly enhanced the accumulation of soluble protein and soluble sugar in host plants.

### 3.7. Detection of Growth-Promoting Functions of QYN6 and Its Effects on Plant Growth

#### 3.7.1. Results of Growth-Promoting Function Detection

**Figure 8:**
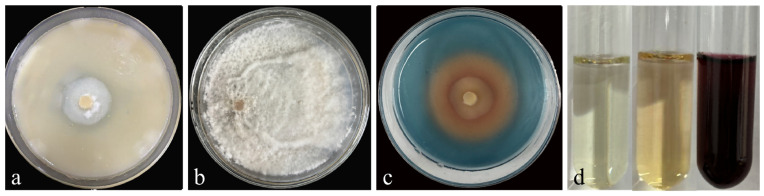
Determination of plant growth-promoting traits of strain QYN6. (**a**) Phosphate-solubilizing ability; (**b**) potassium-solubilizing ability; (**c**) siderophore production; (**d**) IAA-producing ability (from left to right: sterile water, King’s medium, and QYN6 treatment).

The endophytic fungus formed a transparent zone on the organic phosphorus egg yolk medium, indicating it exhibited a certain organic phosphorus-solubilizing ability (Figure 8a). On the potassium-solubilizing detection plate, the strain grew toward potash feldspar powder and even covered it, indicating strong potassium-solubilizing ability (Figure 8b). On the CAS plate, QYN6 clearly produced large quantities of siderophores, which degraded the blue complex formed by CAS and CTAB (Figure 8c). In the IAA secretion assay, significant darkening of the culture was observed, with the liquid approaching purple (Figure 8d). Quantitatively, IAA content was 21.91 μg·mL^−1^, confirming its IAA-secreting ability.

#### 3.7.2. Effects of Strain QYN6 on the Growth of *B. benariensis*

**Figure 9:**
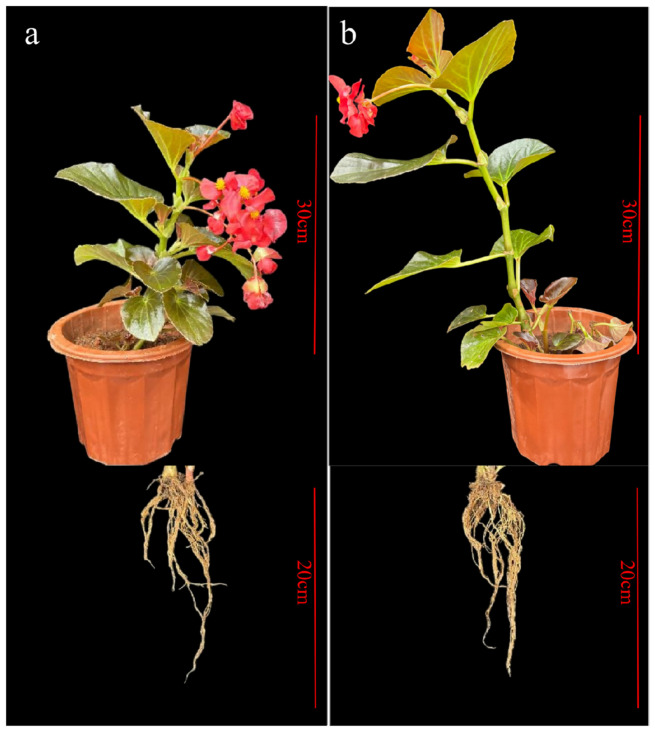
Growth-promoting effect of QYN6 on *B. benariensis*. (**a**) Control group; (**b**) QYN6 treatment group. Note: The scale bar for the potted plant images is 30 cm, and that for the root images is 20 cm.

Thirty days after irrigating *B. benariensis* plants with 100 mL of endophytic fungal suspension, the plants in the endophytic fungus-irrigated treatment group grew relatively better compared with the control group (Figure 9). Across treatments, plant height increment, leaf number increment, root length, shoot fresh weight, shoot dry weight, root fresh weight, and root dry weight of *B. benariensis* in the endophytic fungus-irrigated group were significantly higher than those in the control group (Table 6). These results indicate that the endophytic fungus QYN6 has a positive effect on the growth of *B. benariensis* plants.

## 4. Discussion

In recent years, endophytic fungi have become a research hotspot due to their crucial role in plant growth, defense, and the maintenance of ecosystem functions. Furthermore, this research has led to the development of biological control methods that utilize plant endophytic fungi for preventing and controlling plant diseases [43]. Nutrient and spatial competition is a common antagonistic mode between endophytes and pathogens. Pathogens fail to grow normally when deprived of sufficient nutrients, so dual-culture inhibition capacity can serve as a primary index for screening antagonistic strains [44,45]. In this study, healthy *B*. *benariensis* plants were used as materials, and 31 strains of endophytic fungi were isolated from their roots, stems, and leaves. Among them, four strains exhibited high inhibitory effects, namely QJN4, QYN6, QYN9, and QYN12, with inhibition rates of 55.06%, 63.67%, 57.78%, and 56.65% respectively. QYN6, the most inhibitory strain, was identified as *N. sphaerica*. De Queiroz Brito et al. [46] noted that the genus *Nigrospora* has a broad host range and occurs as saprophytes, endophytes, or pathogens. Importantly, several *Nigrospora* species, including *N. sphaerica*, have been recognized as opportunistic pathogens, capable of causing disease in plants under stress or in immunocompromised human hosts. Wang et al. [47] further emphasized that pathogenicity in *Nigrospora* is strain-specific and strongly influenced by host susceptibility and environmental conditions. While some *N. sphaerica* strains have been reported to cause leaf diseases in crops such as olive [48] and camellia plants [49], our pathogenicity assays showed that QYN6 inoculation did not induce any disease symptoms on healthy *B. benariensis* under normal growth conditions, indicating its low virulence to the host. This is consistent with the view that most endophytic *Nigrospora* isolates, including QYN6, can colonize hosts asymptomatically under normal conditions. Ahmed et al. [50] isolated *N. sphaerica* strains from the leaves of healthy *Pluchea dioscoridis*, and artificial inoculation tests verified that these strains could successfully colonize the intercellular spaces of host leaves without inducing any disease symptoms such as leaf spots, chlorosis, or wilting. Meanwhile, they could secrete antimicrobial substances and show significant inhibitory effects against various plant pathogens, clearly confirming their non-pathogenicity as endophytes and their biocontrol potential. In addition, this strain exerted no adverse impacts on the growth and development of medicinal plant *Euphorbia hirta* L. Its metabolites also had strong antioxidant activity, further supporting the characteristics of *N. sphaerica* as a non-pathogenic endophyte [51]. Nevertheless, its potential risk as an opportunistic pathogen cannot be entirely excluded, especially under adverse environments or host stress. Therefore, further field trials and long-term monitoring are needed to comprehensively evaluate its ecological safety before large-scale application.

Biocontrol fungi achieve biological control of pathogens through specific parasitic behaviors. This hyperparasitic effect supports the development of sustainable plant protection schemes and holds substantial application value for plant disease management systems [52]. Bilański et al. [53] isolated endophytic fungi from *Fraxinus* petioles, with strong antagonistic activity toward *Fraxinus* decline pathogens. Morphological and physiological deformations of the pathogenic hyphae were observed. Some endophytic fungi also attacked pathogenic hyphae via hyperparasitism. Notably, fungal biocontrol against phytopathogens is commonly mediated by synergistic effects of hyperparasitic behavior and CWDEs. For example, in the research by Zhang et al. [54] on the biological control of potato leaf spot disease, the endophytic fungus *Talaromyces muroii* SD1-4 exhibited strong antifungal activity against the pathogen *Alternaria alternata*, causing the pathogenic hyphae to grow in parallel, entangle, and deform. Meanwhile, co-culture of the two fungi significantly increased chitinase and β-1,3-glucanase activities. Similarly, in this study, QYN6 showed hyperparasitism against the pathogen, causing abnormal curling, partial swelling, folding, or partial dissolution and fragmentation of the pathogenic hyphae. Combining the morphological observation and CWDE activity determination, we conclude that CWDE secretion is the predominant antagonistic mechanism of QYN6, while direct hyperparasitism serves as an auxiliary synergistic strategy. QYN6 secreted four CWDEs, among which β-1,3-glucanase, cellulase, and protease were highly expressed. The β-1,3-glucanase activity reached 691.54 U·mL^−1^, which was markedly higher than that of cellulase, protease, and chitinase. Since β-1,3-glucan serves as the major structural skeleton of fungal cell walls [55], the robust β-1,3-glucanase activity of QYN6 confers a core competitive advantage, enabling efficient hydrolysis of the *C. aotearoa* cell wall, disruption of wall integrity, protoplast leakage, and eventual hyphal fragmentation. Cellulase and protease further degrade auxiliary cell wall components, while chitinase plays a minor role, consistent with its low activity. In contrast, hyphal entanglement and curling represent secondary contact interactions that help QYN6 occupy ecological niches to suppress pathogen growth. Notably, no transparent halo was observed on the chitinase plate, which can be explained by the inherent limitations of plate-based detection. Solid plates preferentially visualize high-level enzyme diffusion and fail to reflect low-level or constitutive chitinase secretion. Our supplementary quantitative fermentation assay compensated for this qualitative limitation and accurately confirmed the full CWDE-producing capacity of QYN6.

Biotic and abiotic stresses induce excessive reactive oxygen species (ROS) accumulation in plants, causing oxidative cell membrane damage [56]. Antioxidant enzymes (SOD, PPO, POD, and CAT) and osmotic regulators (soluble proteins and sugars) collectively alleviate ROS-induced injury, stabilize membrane integrity, and enhance plant stress resistance by scavenging toxic ROS, regulating redox homeostasis, and improving osmotic adaptation [57,58,59,60,61,62]. In this study, pathogen infection, QYN6 colonization, and their combined treatment significantly increased the activities of defensive enzymes and the contents of soluble protein and sugar in *B. benariensis* (Figure 7). In particular, SOD and POD activities exhibited a typical two-phase change with an initial decline followed by a sharp increase. This dynamic response reflects a typical biphasic defense strategy during endophyte establishment: transient suppression of host basal immunity at the early stage favors endophyte colonization, whereas stable late colonization activates plant systemic resistance and improves stress adaptability [63,64]. Such immune modulation, coupled with the growth-promoting traits of QYN6, substantially optimizes host defense and metabolic competence against pathogen invasion.

Although soil contains abundant mineral nutrients (e.g., nitrogen, phosphorus, potassium), most exist as insoluble complex salts, limiting direct uptake by plant roots [65,66]. Efficiently activating soil-fixed mineral nutrients and converting them into plant-available forms is a frontier in agricultural research [67,68]. Recent studies have found that certain beneficial microorganisms in the plant rhizosphere possess significant nutrient-degrading capabilities. They activate phosphorus and release potassium by secreting substances (e.g., organic acids). This biological activation not only enhances soil nutrient availability but also promotes plant growth via increased nutrient uptake, which provides sufficient material basis for the synthesis of antioxidant enzymes, soluble sugars, and soluble proteins, thereby facilitating the synthesis and accumulation of plant secondary metabolites and improving plant stress resistance [69,70]. In addition, endophytic fungi can produce siderophores to bind iron ions in the environment for plant uptake, and the secreted IAA can also stimulate plant growth and further regulate host immune and antioxidant metabolic pathways [71].

Lu et al. [72] isolated 113 endophytic fungal strains from *Cotoneaster multiflorus* and screened 25 drought-tolerant strains under simulated drought. Analysis of 12 strains with superior drought tolerance revealed that 7 exhibited significantly higher phosphorus-solubilizing capacity than controls, 6 had potassium-solubilizing ability, and 6 produced IAA, indicating that these endophytes play roles in promoting plant growth and drought tolerance. In the present study, quantitative detection confirmed that QYN6 had siderophore activity and produced IAA at a concentration of 21.91 μg·mL^−1^, demonstrating its excellent nutrient activation and growth-regulating potential. Correspondingly, growth indicator measurements of *B. benariensis* under different treatments showed that plants inoculated with QYN6 exhibited markedly greater increases in plant height and leaf number, longer root length, and higher shoot and root biomass compared with the water-irrigated control group. The improved growth performance and nutrient status of host plants are key to enhancing their antioxidant defense system, enabling plants to better cope with ROS bursts and oxidative damage under pathogen stress.

Notably, QYN6 exhibited a slightly higher biocontrol efficacy against anthracnose caused by *C. aotearoa* in greenhouse pot assays (48.91%) than in detached leaf assays (42.69%). Typically, simplified in vitro conditions tend to yield stronger inhibitory effects [73]. This moderate in vivo efficacy is comparable to previous reports in other plant–pathogen systems, such as endophytic *Bacillus amyloliquefaciens* AsL-1, which achieved a 47.78% inhibition rate against *C. gloeosporioides* in vitro [74]. The satisfactory control effect in potted plants may be attributed to the multiple growth-promoting and immune-activating characteristics of QYN6. Unlike detached leaf systems, which rely solely on direct antagonism, intact living plants can mount induced systemic resistance. QYN6 secretes IAA, produces siderophores and solubilizes nutrients, which continuously optimize plant nutrition and activate systemic defense responses in host tissues. These combined advantages enhance plant adaptability and disease resistance under complex conditions, and consequently improve the biocontrol performance of the strain.

Overall, the endophytic fungus QYN6 inhibits the anthracnose pathogen SWBG5 through multiple mechanisms: hyperparasitizing, CWDE production, host resistance induction, and plant growth promotion. These characteristics indicate its considerable biocontrol potential, making it a promising candidate for the development of biocontrol agents.

## 5. Conclusions

Strain QYN6 produced multiple cell wall-degrading enzymes including chitinase, β-1,3-glucanase, cellulase, and protease, and effectively inhibited the pathogen growth by destroying cell wall structure. It reduced the anthracnose incidence of *B. benariensis* with a control efficacy of 48.91%. Meanwhile, QYN6 displayed excellent plant growth-promoting performances in phosphorus solubilization, potassium release, siderophore production and IAA secretion, which significantly improved plant height, leaf number, root length and biomass. Moreover, QYN6 markedly enhanced plant disease resistance by increasing the activities of leaf antioxidant enzymes (SOD, POD, CAT and PPO). Collectively, QYN6 possesses outstanding biocontrol and growth-promoting potentials, and can serve as a promising candidate strain for the development of biocontrol agents against *B. benariensis* anthracnose.

In future studies, high-throughput sequencing technology will be employed to analyze the effects of strain QYN6 on rhizosphere microbial diversity, community structure, and functions of *B. benariensis*. Meanwhile, transcriptomic and metabolomic analyses will be utilized to elucidate the underlying response mechanisms of the interaction between QYN6 and its host plant, and to further explore the biocontrol and growth-promoting mechanisms of this strain in *Begonia* species as well as other herbaceous plants. Although the strain exhibited favorable antagonistic and growth-promoting properties, further research is needed to explore its formulation stability, environmental adaptability, and potential obstacles to commercial application. In addition, more verification tests against different pathogenic fungi should be carried out.

## Figures and Tables

**Table 1:** The primer information and PCR amplification program used in this study.

Locus	Primer Name	Sequence (5′-3′)	PCR Amplification Program
*ITS*	ITS1	TCCGTAGGTGAACCTGCGG	95 °C pre-denaturation for 5 min; 35 cycles of 94 °C for 30 s, 52 °C for 45 s, 72 °C for 50 s; 72 °C extension for 10 min
	ITS4	TCCTCCGCTTATTGATATGC	
*TUB2*	Bt2a	GGTAACCAAATCGGTGCTGCTTTC	95 °C pre-denaturation for 2 min; 35 cycles of 95 °C for 30 s, 60 °C for 30 s, 72 °C for 30 s; 72 °C extension for 10 min
	Bt2b	ACCCTCAGTGTAGTGACCCTTGGC	
*TEF-1α*	EF1-728F	CATCGAGAAGTTCGAGAAGG	95 °C pre-denaturation for 5 min; 30 cycles of 95 °C for 30 s, 55 °C for 30 s, 72 °C for 1 min; 72 °C extension for 10 min
	EF1-986R	TACTTGAAGGAACCCTTACC	

**Table 2:** PCR reaction system for *ITS* and *TUB2* genes.

Reagent	Volume (μL)
ddH_2_O	11
2 × Taq PCR Master Mix	10
Primer-F	1
Primer-R	1
Template	2
total volume	25

**Table 3:** PCR reaction system for *TEF-1α* gene.

Reagent	Volume (μL)
ddH_2_O	7
2 × Taq PCR Master Mix	10
Primer-F	1
Primer-R	1
Template	1
total volume	20

**Table 4:** Inhibition rate of four endophytic fungi on anthracnose pathogen in *B. benariensis*.

Strains	Inhibition Rate (%)
QJN4	55.06 ± 1.70 d
QYN6	63.67 ± 1.61 a
QYN9	57.78 ± 0.14 b
QYN12	56.65 ± 0.94 c

Note: Different lowercase letters indicate significant differences at *p* < 0.05.

**Table 5:** Biocontrol efficacy of strain QYN6 against anthracnose caused by SWBG5.

	In Vitro Biocontrol Efficacy		Potted Plant Biocontrol Efficacy	
	Disease Index	Biocontrol Efficacy (%)	Disease Index	Biocontrol Efficacy (%)
CK	72.22 ± 4.81 a		31.81 ± 5.90 a	
QYN6	41.67 ± 8.50 b	42.69 ± 8.78	16.25 ± 3.31 b	48.91 ± 13.47

Note: Different lowercase letters indicate significant differences at *p* < 0.05.

**Table 6:** Effect of QYN6 on the growth of *B. benariensis*.

Treatment	Plant Height Increment (cm)	Leaf Number Increment (Pieces)	Root Length (cm)	Shoot Fresh Weight (g)	Shoot Dry Weight (g)	Root Fresh Weight (g)	Root Dry Weight (g)
Control	9.30 ± 0.26 b	6.67 ± 0.58 b	17.57 ± 0.40 b	62.14 ± 1.64 b	3.20 ± 0.37 b	2.96 ± 0.58 b	0.55 ± 0.02 b
QYN6	21.27 ± 1.51 a	8.00 ± 2.00 a	20.63 ± 0.35 a	98.78 ± 0.65 a	4.37 ± 0.04 a	5.26 ± 1.18 a	0.88 ± 0.08 a

Note: Different lowercase letters indicate significant differences at *p* < 0.05.

## Data Availability

All sequences of the strain have been uploaded to NCBI and the accession numbers are PV336075 (*ITS*), PV363142 (*TUB2*), PV363145(*TEF-1α*).

## Supplementary Materials

The following supporting information can be downloaded at: https://www.mdpi.com/article/10.3390/jof12060412/s1.
