# Supplementary material for: Biological Control and Growth-Promoting Potential of the Endophytic Fungus Nigrospora sphaerica Against Anthracnose in Begonia benariensis
Source: J Fungi (Basel). 2026 Jun 5;12(6):412. doi: 10.3390/jof12060412 (PMC13301808; doi:10.3390/jof12060412)
Supplement: Supplementary file 1 [file jof-12-00412-s001.zip › jof-4324882-supplementary.pdf]

supplementary materials

1 Medium

Table S1 Medium Formulation

Medium Name	Formulation
Potato Dextrose Agar Medium(PDA)	Potato 200 g, agar 15–20 g, glucose 20 g, distilled water 1000 mL, natural pH.
Chitinase Qualitative Medium	1% colloidal chitin 3 g, KH_2PO_4 0.4 g, $MgSO_4$ 0.6 g, K_2HPO_4 0.6 g, $FeSO_4$ 0.02 g, distilled water 1000 mL, pH 7.0
β-1,3-Glucanase Qualitative Medium	β-1,3-glucan 5 g, $NaNO_3$ 2 g, K_2HPO_4 1 g, KCl 0.5 g, $MgSO_4$ 0.5 g, $FeSO_4$ 0.01 g, Congo red 0.05 g, agar 15–20 g, distilled water 1000 mL, pH 7.0.
Cellulase Qualitative Medium	Sodium carboxymethyl cellulose 10 g, K_2HPO_4 2 g, $MgSO_4$ 1 g, NaCl 1 g, Congo red 0.1 g, agar 15–20 g, distilled water 1000 mL, pH 6.0.
Protease Qualitative Medium	Beef extract 1 g, peptone 0.5 g, NaCl 0.5 g, casein 1 g, agar 15–20 g, distilled water 1000 mL, pH 7.0.
Chitinase Quantitative Medium	Chitinase qualitative medium without agar
β-1,3-Glucanase Quantitative Medium	β-1,3-glucanase qualitative medium without agar
Cellulase Quantitative Medium	Sodium carboxymethyl cellulose 10 g, yeast extract 8 g, K_2HPO_4 3 g, $(NH_4)_2SO_4$ 2 g, $CaCO_3$ 2 g, glucose 1.5 g, $MgSO_4$ 0.5 g, distilled water 1000 mL, pH 6.0.
Protease Quantitative Medium	Peptone 0.5 g, casein 0.8 g, yeast extract 0.1 g, glucose 0.1 g, NaCl 0.58 g, anhydrous sodium citrate 0.45 g, distilled water 1000 mL, pH 7.0.
Organophosphorus Egg Yolk Medium	NaCl 5 g, beef extract 5 g, peptone 10 g, agar 15–20 g, distilled water 1000 mL, pH 7–7.5. When in use, add 3 mL of fresh egg yolk solution (sterile physiological saline: egg yolk = 1:1) to every 50 mL of the medium.
King's B Medium	Peptone 10 g, $MgSO_4$ 1.5 g, K_2HPO_4 0.5 g,

	glycerol 10 mL, distilled water 1000 mL.
Chrome Azurol S Medium(CAS)	Chrome azurol S medium powder 10.87 g, distilled water 1000 mL
Iron-Free Czapek Medium	NaCl 0.5 g, $NaNO_3$ 2 g, K_2HPO_4 1 g, $MgSO_4$ 0.5 g, sucrose 30 g, distilled water 1000 mL.

2 Reagent Formulation

(1) Phosphate buffer: Weigh 71.6 g of $Na_2HPO_4·12H_2O$, dissolve it in a small amount of deionized water, and dilute to 1000 mL; this is referred to as "0.2 mol/L Solution A". Weigh 31.2 g of $NaH_2PO_4·2H_2O$, dissolve it in a small amount of deionized water, and dilute to 1000 mL; this is referred to as "0.2 mol/L Solution B". Dilute Solution A and Solution B as required, then prepare the desired pH according to Table 1.

Table S2 Preparation of phosphate buffer solutions with different pH values(mL)

pH	A	B		pH	A	B		pH	A	B		pH	A	B
5.7	6.5	93.5		6.3	22.5	77.5		6.9	55.0	45.0		7.5	84.0	16.0
5.8	8.0	92.0		6.4	26.5	73.5		7.0	62.0	38.0		7.6	87.0	13.0
5.9	10.0	90.0		6.5	31.5	68.5		7.1	67.0	33.0		7.7	89.5	10.5
6.0	12.3	87.7		6.6	37.5	62.5		7.2	72.0	28.0		7.8	91.5	8.5
6.1	15.0	85.0		6.7	43.5	56.5		7.3	77.0	23.0		7.9	93.0	7.0
6.2	18.5	81.5		6.8	49.0	51.0		7.4	81.0	19.0		8.0	94.7	5.3

(2)1% colloidal chitin: Accurately weigh 1 g of chitin into 20 mL of concentrated hydrochloric acid, stir in an ice bath under ventilation until the precipitate is dissolved, and place at 4°C for 24 h. After stratification, collect the supernatant, add 100 mL of 50% ethanol, stir thoroughly, centrifuge at 8000 r/min for 15 min to remove the supernatant, and repeatedly rinse with deionized water until the pH reaches approximately 7. Finally, dilute to 100 mL with 0.1 mol/L phosphate buffer (pH 6.0) to obtain 1% colloidal chitin, which is stored at 4°C for later use.

(3)N-acetylglucosamine standard solution: Place the N-acetylglucosamine standard in an oven at 80°C and dry to constant weight. Accurately weigh 1 g of it, dissolve in 100 mL of deionized water, and prepare a standard solution with a concentration of 1 mg/mL.

(4)Glucose standard solution: Place the anhydrous glucose standard in an oven at 80°C and bake to constant weight. Accurately weigh 1 g of it, dissolve in 100 mL of deionized water, and prepare a standard solution with a concentration of 1 mg/mL.

(5)1% glucan: Place the β-1,3-glucan standard in an oven at 80°C and dry to constant weight. Accurately weigh 1 g of it, dissolve in phosphate buffer (0.1 mol/L, pH 6.0), and dilute to 100 mL.
(6)0.1 g/mL sodium carboxymethyl cellulose (CMC-Na): Place the sodium carboxymethyl cellulose standard in an oven at 80°C and dry to constant weight. Accurately weigh 1 g of it. Heat deionized water to 60°C, add the drug in small amounts multiple times, stir while adding to prevent agglomeration, and stir thoroughly to form a paste-like glue. Dilute to 100 mL and let it stand for later use.
(7)NaOH solution (2 mol/L): Weigh 40 g of NaOH standard, dissolve it in an appropriate amount of deionized water, and after cooling, dilute to 500 mL with deionized water.
(8)3,5-dinitrosalicylic acid reagent (DNS): Weigh 6.3 g of 3,5-dinitrosalicylic acid, 262 mL of 2 mol/L NaOH, 5 g of crystalline phenol, and 5 g of Na_2SO_3, and sequentially dissolve them in a 500 mL hot aqueous solution containing 185 g of potassium sodium tartrate. Stir to ensure complete dissolution, cool to room temperature, add deionized water to dilute to 1000 mL, store in a brown ground-glass bottle, and let it stand in a cool place for 7 days before use. If turbidity occurs during use, it can be used after washing.
(9)HCl solution (1 mol/L): Pipette 8.3 mL of concentrated hydrochloric acid, add it to deionized water in small amounts multiple times, stir and cool, then dilute to 100 mL with deionized water.
(10)Tyrosine standard solution: Dry tyrosine in an oven at 80°C to constant weight, accurately weigh 1 g of it, dissolve it in a small amount of 1 mol/L HCl solution, and dilute to 100 mL to obtain a standard solution with a concentration of 1 mg/mL.
(12)1% casein solution: Accurately weigh 1 g of casein, mix it with a small amount of deionized water and 0.5 mol/L NaOH solution, then heat in a microwave oven until dissolved. After cooling to room temperature, dilute to 100 mL. Store at 4°C and prepare fresh as needed.
(13)Trichloroacetic acid solution (0.4 mol/L): Dissolve 6.54 g of trichloroacetic acid completely in 50 mL of deionized water, and dilute to 100 mL.
(14)Sodium carbonate solution (0.4 mol/L): Dissolve 42.4 g of Na_2CO_3 completely in 500 mL of deionized water, and dilute to 1000 mL.
(15) CAS detection solution: Accurately weigh 0.079 g of chrome azurol S an d dissolve it in 50 mL of deionized water. Then add 10 mL of 1 mmol/L Fe Cl_3 to the solution, which is referred to as "Solution A". Accurately weigh ano ther 0.069 g of cetyltrimethylammonium bromide (CTAB) and dissolve it in 40

mL of deionized water, which is referred to as "Solution B". Subsequently, slowly add Solution A to Solution B while stirring uniformly.

(16) IAA solution (100 μg/mL): Accurately weigh 10 mg of IAA, first dissolve it in a small amount of ethanol, and then dilute to 100 mL with distilled water.

(17) Salkowski chromogenic solution (IAA measurement range: 5–200 mg/L): Dissolve 4.5 g of $FeCl_3$ in 300 mL of deionized water, then slowly add 587.4 mL of concentrated sulfuric acid to the solution. The addition of concentrated sulfuric acid will generate heat; after the solution cools down, dilute to 1000 mL with deionized water.

(18) Methionine (Met) solution (130 mmol/L): Dissolve 1.9399 g of Met in phosphate buffer (0.05 mol/L, pH 7.8), dilute to 100 mL, and store at 4°C in the dark.

(19) Nitroblue tetrazolium (NBT) solution (750 μmol/L): Dissolve 0.06134 g of NBT in phosphate buffer (0.05 mol/L, pH 7.8), dilute to 100 mL, and store at 4°C in the dark.

(20) Disodium ethylenediaminetetraacetate (EDTA-2Na) solution (100 μmol/L): Weigh 0.0093 g of EDTA-2Na and dilute to 250 mL with deionized water.

(21) Riboflavin solution (20 μmol/L): Weigh 0.03765 g of riboflavin, dilute to 500 mL with deionized water, and store at low temperature in the dark.

(22) Guaiacol solution (0.05 mol/L): Pipette 5.61 mL of guaiacol stock solution (purity ≥ 98%), dilute and dissolve it in a small amount of ethanol, then dilute to 250 mL with deionized water.

(23) Catechol solution (0.01 mol/L): Dissolve 0.55 g of catechol in deionized water, dilute to 500 mL, and store in a brown volumetric flask for later use.

(24) 1% polyvinylpyrrolidone (PVP) phosphate buffer: Dissolve 1 g of PVP in phosphate buffer (0.1 mol/L, pH 7.0) and dilute to 100 mL.

(25) Standard bovine serum albumin solution (100 μg/mL): Accurately weigh 0.01 g of bovine serum albumin, dissolve it in distilled water, and dilute to 100 mL.

(26) Coomassie brilliant blue G-250 solution: Dissolve 0.1 g of Coomassie brilliant blue G-250 in 50 mL of 90% ethanol, then add 100 mL of 85% phosphoric acid, dilute to 1000 mL, transfer to a brown bottle, and store at room temperature (valid for 1 month).

(27) Phenol solution (90 μg/mL): Take 9 g of crystalline phenol, dissolve it in a small amount of deionized water, and dilute to 100 mL.

(28) Sucrose standard solution (100 μg/mL): Dry sucrose in an oven at 80°C to constant weight, accurately weigh 1 g of it, dissolve in a small amount of deionized water, add 0.5 mL of H_2SO_4 after dissolution, and dilute to 1000 mL.

3 Quantitative detection method for cell wall degrading enzymes

3.1 Quantitative detection of chitinase

Preparation of crude enzyme solution: In a clean bench, transfer the endop hytic fungal cakes into sterilized chitinase quantitative medium, with 3 cakes a dded per 100 mL medium. After the experiment setup, seal the containers and place them in a shaker. Cultivate at 28°C with a rotation speed of 180 r/min for 72 hours, then take out the shake flasks. Centrifuge at 3000 r/min for 15 minutes, and the supernatant is the crude enzyme solution.

Drawing of N-acetylglucosamine standard curve: Add N-acetylglucosamine standard solution into test tubes with volumes of 0, 0.25, 0.30, 0.40, 0.45, 0.5 0, and 0.55 mL respectively, and make up to 1 mL with distilled water. Then add 1.5 mL of DNS reagent, and finally add 3.5 mL of distilled water to dil ute to a total volume of 7.5 mL. Preheat the spectrophotometer for 30 minutes, set the zero with distilled water, and measure the absorbance at 540 nm. Dra w the standard curve with absorbance as the ordinate and N-acetylglucosamine concentration as the abscissa.

Determination of enzyme activity: Mix 0.5 mL of crude enzyme solution with 0.5 mL of phosphate buffer (0.1 mol/L, pH 6.0) containing 1% colloidal chitin, incubate at 50°C for 30 minutes, centrifuge at 10,000 r/min for 10 min utes. Take the supernatant and add 1 mL of DNS reagent, place in a boiling water bath at 100°C for 5 minutes, and dilute to 7.5 mL after cooling. Each measurement is repeated 3 times, and the absorbance is determined at a wavel ength of 540 nm. The enzyme solution inactivated by boiling water bath at 10 0°C for 15 minutes is used as the standard control. The enzyme activity unit i s defined as the amount of enzyme required for 1 mL of enzyme solution to catalyze the substrate to produce 1 μg of reducing sugar per minute when the substrate reacts with the crude enzyme solution at 50°C and pH 6.0 for 30 mi nutes.

Enzyme activity(U/mL·min) =(X×V_total×1000)/(V_sample×T)

Note: x represents the reducing sugar content calculated by substituting the me asured absorbance of the sample into the standard curve formula; V_total is th e total volume of the measured sample; 1000 is the conversion factor (1 mg = 1000 μg); V_sample is the volume of the enzyme solution used for measurem ent; T is the reaction time.

3.2 Quantitative detection of β-1,3-glucanase

Preparation of crude enzyme solution: In a clean bench, transfer the endop hytic fungal cakes into sterilized β-1,3-glucanase quantitative medium, with 3 c akes added per 100 mL medium. After the experiment setup, seal the container s and place them in a shaker. Cultivate at 28°C with a rotation speed of 180 r/min for 120 hours, then take out the shake flasks. Centrifuge at 8000 r/min for 10 minutes, and the supernatant is the cru de enzyme solution.

Drawing of glucose standard curve: Prepare a series of working solutions of glucose standard solution with concentrations of 0.4, 0.8, 1, 1.2, 1.6, and 2 mg/mL. Add 2 mL of each solution to test tubes in sequence, then add 3 m L of DNS reagent, and finally add 20 mL of distilled water to dilute to a tota l volume of 25 mL. Preheat the spectrophotomete for 30 minutes, set the zero with distilled water, and measure the absorbance at 540 nm. Draw the standar d curve with absorbance as the ordinate and glucose concentration as theabscis sa.

Determination of enzyme activity: Mix 0.5 mL of crude enzyme solution with 0.5 mL of phosphate buffer (0.1 mol/L, pH 6.0) containing 1% glucan un iformly, incubate at 50°C for 30 minutes, then cool. Add 3 mL of DNS reage nt, place in a boiling water bath at 100°C for 5 minutes, and dilute to 25 mL after cooling. Measure the absorbance at a wavelength of 540 nm. The enzym e solution inactivated by boiling water bath at 100°C for 1
minutes is used as the standard control. The enzyme activity unit is defined as the amount of enzyme required for 1 mL of enzyme solution to catalyze the substrate to produce 1 μg of reducing sugar per minute when the substrate rea cts with the crude enzyme solution at 50°C and pH 6.0 for 30 minutes. The c alculation formula for β-1,3-glucanase activity is the same as that for chitinase activity.

3.3 Quantitative detection of cellulase

Preparation of crude enzyme solution: In a clean bench, transfer the endophytic fungal caes into sterilized cellulase quantitative medium, with 3 cakes added per 100 mL medium.After the experiment setup, seal the containers and place them in a shaker. Cultivate at 8°C with a rotation speed of 180 r/min for 120 hours, then take out the shake flasks. Centrifuge at 8000 r/min for 10 minutes, and the supernatant is the crude enzyme solution.

Drawing of glucose standard curve: Same as the standard curve for β-1,3-glucanase.

Determination of enzyme activity: Mix 0.5 mL of crude enzyme solution with 0.5 mL of phosphate buffer (0.1 mol/L, pH 6.0) containing 1% glucan un iformly, incubate at 50°C for 30 minutes, then cool. Add 3 mL of DNS reage nt, place in a boiling water bath at 100°C for 5 minutes, and dilute to 25 mL after cooling. Measure the absorbance at a wavelength of 540 nm. The enzym e solution inactivated by boiling water bath at 100°C for 15 minutes is used a s the standard control. The enzyme activity unit is defined as the amount of e nzyme required for 1 mL of enzyme solution to catalyze the substrate to prod uce 1 μg of reducing sugar per minute when the substrate reacts with the crud

e enzyme solution at 50°C and pH 6.0 for 30 minutes. The calculation formula for cellulase activity is the same as that for chitinase activity.

3.4 Quantitative detection of protease

Preparation of crude enzyme solution: In a clean bench, transfer the endophytic fungal cakes into sterilized protease quantitative medium, with 3 cakes added per 100 mL medium. After the experiment setup, seal the containers and place them in a shaker. Cultivate at 35°C with a rotation speed of 200 r/min for 120 hours, then take out the shake flasks. Centrifuge at 8000 r/min for 10 minutes, and the supernatant is the crude enzyme solution.

Drawing of tyrosine standard curve: Add 0.0, 0.2, 0.4, 0.5, 0.6, 0.8, and 1.0 mL of 0.1 mg/mL tyrosine standard solution respectively, then add distilled water to each test tube to make the total volume 1.0 mL. Subsequently, add 5 mL of 0.4 mol/L Na_2CO_3 solution and 1 mL of Folin-Ciocalteu reagent to each tube. After thorough oscillation, incubate in a 40°C water bath for 20 minutes. Shake the solution in the colorimetric tube, determine the absorbance of tyrosine at different concentrations at a wavelength of 680 nm, and draw the standard curve with absorbance as the ordinate and tyrosine concentration as the abscissa.

Determination of enzyme activity: Add 0.5 mL of crude enzyme solution and 1 mL of 1% casein solution to a test tube in sequence, and incubate in a 40°C water bath for 20 minutes. Take it out and cool, then add 2 mL of 0.4 mol/L trichloroacetic acid solution, place it in a 40°C water bath again for 15 minutes, then centrifuge at 3000 r/min for 5 minutes. Discard the lower precipitate, take 1 mL of supernatant, add 5 mL of 0.4 mol/L Na_2CO_3 solution and 1 mL of Folin-Ciocalteu reagent in sequence, invert to mix, develop color in a 40°C water bath for 20 minutes, and finally determine the absorbance at a wavelength of 680 nm. The enzyme activity unit is defined as the amount of enzyme required for 1 mL of enzyme solution to catalyze the substrate to produce 1 μg of tyrosine per minute when the substrate reacts with the crude enzyme solution at 40°C for 20 minutes. Its activity calculation formula is the same as that for chitinase activity.

4 Determination of activities of several defense enzymes and contents of soluble sugar and soluble protein

4.1 Determination of SOD enzyme activity

(1) Extraction of enzyme solution from fresh samples: Mix 0.5 g of fresh sample with

1 mL of pre-cooled phosphate buffer (0.05 mol/L, pH 7.8), grind in an ice bath in a pre-cooled mortar until the fresh sample becomes a homogenate. Transfer the homogenate to a 10 mL centrifuge tube using a pipette, then wash the mortar with 4 mL of buffer, and transfer the washing solution to the centrifuge tube; the total volume of the extract is 5 mL. Centrifuge at 1000 r/min at 4°C for 20 minutes, and the supernatant is the crude enzyme extract.

(2) Determination of enzyme activity in crude extract: Take 4 test tubes, labeled as 0 (CK), 1, 2, and 3. Add different solutions to the test tubes in sequence according to Table 6, mix well, then place tubes 1, 2, and 3 under 4000 lx at 25°C for light color development for 20 minutes, while tube 0 is not exposed to light.

Table S3 SOD enzyme reaction liquid system(mL)

buffer solution(0.05 mol/L pH 7.8)	Met	NBT	EDTA-Na_2	riboflavin	pure water	enzyme solution
1.5	0.3	0.3	0.3	0.3	0.2	0.1

After the reaction, immediately shield from light to prevent the reaction from proceeding. Measure the absorbance of each tube at a wavelength of 560 nm using an ultraviolet spectrophotometer. The SOD enzyme activity is calculated with 1 enzyme activity unit defined as the amount of enzyme that inhibits 50% of NBT photoreduction per gram of sample.

$$\text{SOD activity(U/g·min)}=\frac{(A_{CK}-A)\times V}{50\%\times A_{CK}\times W\times T\times V_t}$$

Note: In the formula, A_ck represents the absorbance measured in the control tube, A represents the absorbance measured in the sample tube, V is the total volume of the extract, W is the weight of the fresh sample, T is the reaction time, and V_t is the volume of the extract used in the reaction.

4.2 Determination of POD enzyme activity

(1) Extraction of enzyme solution from fresh samples: Mix 0.5 g of fresh sample with 2.5 mL of pre-cooled phosphate buffer (0.1 mol/L, pH 6.0), grind in an ice bath in a pre-cooled mortar until the fresh sample becomes a homogenate. Transfer the homogenate to a 10 mL centrifuge tube using a pipette, then wash the mortar with 2.5 mL of buffer, and transfer the washing solution to the centrifuge tube; the total volume of the extract is 5 mL. Centrifuge at 8000 r/min for 10 minutes, collect the supernatant into a 10 mL centrifuge tube, and store at 4°C for later use.

(2) Preparation of reaction mixture: Add 112 μL of guaiacol to 200 mL of phosphate buffer solution (0.1 mol/L, pH 6.0), heat and stir. After the solution c

ools down, add 76 μL of 30% H_2O_2, mix uniformly, and store at 4°C for late
r use.

(3) Determination of enzyme activity in crude extract: Take 4 test tubes, labele
d 0 to 3, where tube 0 serves as the zero-calibration control and the other thr
ee are sample tubes. Add 3 mL of reaction mixture and 0.1 mL of enzyme so
lution to each test tube (measure immediately after addition). For the control, r
eplace the enzyme solution with buffer. Measure the absorbance at a wavelengt
h of 470 nm, take 3 readings with an interval of 30 seconds between each rea
ding. The POD enzyme activity is calculated with 1 enzyme activity unit defin
ed as a 0.01 change in A_{470} per minute per gram of sample.

$$\text{POD activity(U/g·min)}=\frac{\Delta A470\times V}{0.01\times W\times T\times V_t}$$

In the formula, ΔA represents the change in absorbance between the initial and
final stages of the reaction; V is the total volume of the extract; W is the w
eight of the fresh sample; T is the reaction time; and Vt is the volume of the
extract used in the reaction.

4.3 Determination of PPO enzyme activity
(1) Extraction of enzyme solution from fresh samples: Mix 0.5 g of fresh sam
ple with 3 mL of pre-cooled 1% PVP phosphate buffer, grind in an ice bath i
n a pre-cooled mortar until the fresh sample becomes a homogenate. Transfer t
he homogenate to a 10 mL centrifuge tube using a pipette, then wash the mor
tar with 7 mL of 1% PVP phosphate buffer, and transfer the washing solution
to the centrifuge tube; the total volume of the extract is 10 mL. Centrifuge a
t 8000 r/min for 5 minutes, collect the supernatant into a 10 mL centrifuge tu
be, and store at 4°C for later use.

(2) Determination of enzyme activity in crude extract: Take 4 test tubes, labele
d 0 to 3. For tubes 1, 2, and 3, add 0.5 mL of catechol, 2.0 mL of phosphat
e buffer (0.1 mol/L, pH 7.0), and 0.5 mL of enzyme solution in sequence (me
asure immediately after addition). For tube 0, 0.5 mL of buffer is used instead
of the enzyme solution as the zero-calibration control. After shaking well, det
ermine the absorbance at a wavelength of 410 nm, take 2 readings with an int
erval of 2 minutes between each reading. The PPO enzyme activity is calculat
ed with 1 enzyme activity unit defined as a 0.01 increase in A_{410} per minute
per gram of sample.

$$\text{PPO activity(U/g·min)}=\frac{\Delta A410\times V}{0.01\times W\times T\times V_t}$$

Note: In the formula, ΔA represents the change in absorbance between the initi
al and final stages of the reaction; V is the total volume of the extract; W is
the weight of the fresh sample; T is the reaction time; and Vt is the volume
of the extract used in the reaction.

4.4 Determination of CAT enzyme activity

(1) Extraction of enzyme solution from fresh samples: Mix 0.5 g of fresh sample with 4 mL of pre-cooled phosphate buffer (0.05 mol/L, pH 8.0), grind in an ice bath in a pre-cooled mortar until the fresh sample becomes a homogenate. Transfer the homogenate to a 10 mL centrifuge tube using a pipette; the total volume of the extract is 4 mL. Centrifuge at 8000 r/min for 15 minutes, collect the supernatant into a 10 mL centrifuge tube, and store at 4°C for later use.

(2) Preparation of reaction mixture: Mix 200 mL of phosphate buffer (0.05 mol/L, pH 8.0) with 0.3092 mL of 30% H_2O_2 uniformly, and store at 4°C for later use.

(3) Determination of enzyme activity in crude extract: Take 3 test tubes, labeled 1 to 3. Add 3 mL of reaction mixture to each test tube, then add 0.1 mL of enzyme solution (measure immediately after addition). Use 3 mL of buffer as the zero-calibration control. After shaking well, determine the absorbance at a wavelength of 240 nm (using a quartz cuvette), take 2 readings with an interval of 1 minute between each reading. The CAT enzyme activity is calculated with 1 enzyme activity unit defined as a 0.01 decrease in A_{240} per minute per gram of sample.

$$\text{CAT activity(U/g·min)}=\frac{\Delta A240\times V}{0.01\times W\times T\times V_t}$$

Note: In the formula, ΔA represents the change in absorbance between the initial and final stages of the reaction; V is the total volume of the extract; W is the weight of the fresh sample; T is the reaction time; and Vt is the volume of the extract used in the reaction.

4.5 Soluble protein content

(1) Drawing of standard curve: Prepare a series of working solutions with concentrations of 0.5, 1, 3, 5, 10, 12.5, and 15 μg/mL using a 100 μg/mL standard bovine serum albumin solution. Take clean test tubes, add 1 mL of the working solutions in order of concentration, then add 5 mL of Coomassie Brilliant Blue G-250 solution, shake well, and let stand for 5 minutes for color development. Determine the absorbance of each tube at a wavelength of 595 nm, and draw the standard curve with the concentration of the series working solutions as the abscissa and the absorbance as the ordinate.

(2) Sample extraction and determination: Mix 0.3 g of fresh sample with 2.5 mL of pure water and grind into a homogenate. Transfer the homogenate to a

10 mL centrifuge tube using a pipette, then rinse the mortar with 2.5 mL of pure water, and pipette the rinsing solution into the centrifuge tube; the total v olume of the extract is 5 mL. Centrifuge at 3000 r/min for 10 minutes, collect the supernatant, and store at 4°C for later use. Mix 1 mL of the extract with 5 mL of Coomassie Brilliant Blue G-250 solution thoroughly, let stand for 5 minutes for color development, then determine the absorbance at a wavelength of 595 nm. The protein concentration is obtained from the standard curve, and the soluble protein content in the sample is calculated.

$$\textit{soluble protein content}(\text{mg/g})=\frac{V\times C}{W\times V_t\times 1000}$$

In the formula, V represents the total volume of the extract; C represents the protein concentration calculated from the standard curve; W represents the weig ht of the fresh sample; and Vt represents the volume of the extract used in th e reaction.

4.6 Determination of soluble sugar content

(1) Drawing of standard curve: Prepare a series of working solutions with conc entrations of 0, 5, 10, 20, 30, 40, 45, and 50 μg/mL using a 100 μg/mL stand ard soluble sugar solution. Take clean test tubes, add 1 mL of the working sol utions in order of concentration, then add 0.5 mL of 90 μg/mL crystalline phe nol and 2.5 mL of H_2SO_4, and let stand for 30 minutes for color development. Determine the absorbance of each tube at 485 nm, and draw the standard cur ve with the concentration of the series working solutions as the abscissa and t he absorbance as the ordinate.

(2) Sample extraction and determination: Mix 0.5 g of fresh sample with 6 m L of pure water and grind into a homogenate. Transfer the homogenate to a 1 0 mL centrifuge tube using a pipette, then rinse the mortar with 4 mL of pure water, and pipette the rinsing solution into the centrifuge tube; the total volu me of the extract is 10 mL. After a boiling water bath for 30 minutes, directl y filter through filter paper into a 100 mL volumetric flask, and repeatedly rin se the filter paper until the solution reaches the scale mark. Then pipette 5 m L of the diluted solution from the volumetric flask into a 25 mL volumetric fl ask and continue to dilute to the scale mark. Mix 1 mL of the final diluted s olution, 0.5 mL of 90 μg/mL crystalline phenol, and 2.5 mL of H_2SO_4 thoroug hly, let stand for 30 minutes for color development, cool to room temperature, and determine the absorbance at 485 nm. The sugar content is found from th e standard curve based on the absorbance, and the soluble sugar content in the sample is calculated.

$$\text{soluble protein content}(\text{mg/g})=\frac{V\times C\times n}{W\times V_t\times 1000}$$

In the formula, V represents the total volume of the extract; C represents the sugar content calculated from the standard curve; n represents the dilution factor; W

represents the weight of the fresh sample; and Vt represents the volume of the extract used in the reaction.
